# Deep Learning-Based Fish Detection Using Above-Water Infrared Camera for Deep-Sea Aquaculture: A Comparison Study

**DOI:** 10.3390/s24082430

**Published:** 2024-04-10

**Authors:** Gen Li, Zidan Yao, Yu Hu, Anji Lian, Taiping Yuan, Guoliang Pang, Xiaohua Huang

**Affiliations:** 1 South China Sea Fisheries Research Institute, Chinese Academy of Fishery Sciences, Guangzhou 510300, China; ligen@scsfri.ac.cn (G.L.); huyu@scsfri.ac.cn (Y.H.); anji_lian@foxmail.com (A.L.); mars@scsfri.ac.cn (T.Y.); pangguoliang@scsfri.ac.cn (G.P.); 2 Key Laboratory of Open-Sea Fishery Development, Ministry of Agriculture and Rural Affairs, Guangzhou 510300, China; 3 Research and Development Center for Tropical Aquatic Products, South China Sea Fisheries Research Institute, Chinese Academy of Fishery Sciences, Sanya 572018, China; 4 Sanya Tropical Fisheries Research Institute, Sanya 572018, China; 5 School of Marine Engineering Equipment, Zhejiang Ocean University, Zhoushan 316022, China; yaozidan@zjou.edu.cn

**Keywords:** fish detection, fish dataset, Faster R-CNN, above-water infrared camera, deep-sea aquaculture

## Abstract

Long-term, automated fish detection provides invaluable data for deep-sea aquaculture, which is crucial for safe and efficient seawater aquafarming. In this paper, we used an infrared camera installed on a deep-sea truss-structure net cage to collect fish images, which were subsequently labeled to establish a fish dataset. Comparison experiments with our dataset based on Faster R-CNN as the basic objection detection framework were conducted to explore how different backbone networks and network improvement modules influenced fish detection performances. Furthermore, we also experimented with the effects of different learning rates, feature extraction layers, and data augmentation strategies. Our results showed that Faster R-CNN with the EfficientNetB0 backbone and FPN module was the most competitive fish detection network for our dataset, since it took a significantly shorter detection time while maintaining a high AP50 value of 0.85, compared to the best AP50 value of 0.86 being achieved by the combination of VGG16 with all improvement modules plus data augmentation. Overall, this work has verified the effectiveness of deep learning-based object detection methods and provided insights into subsequent network improvements.

## 1. Introduction

Seawater aquafarming, characterized by its high quality and large yield, has gradually increased its share in global fishery production in recent years [1,2]. Deep-sea aquaculture, which is a concept originating from the Ministry of Agriculture and Rural Affairs of the People’s Republic of China, refers to seawater aquafarming in marine zones located more than 10 km from the coast and at depths greater than 20 m. To improve efficiency as well as reduce the costs and risks associated with deep-sea aquaculture, automated and online fish detection is one of the key issues that need to be addressed. It not only aids in creating rational feeding schedules and harvesting cycles but also allows for timely recognition of abnormal behaviors of fish schools, which helps to identify changes in water quality and damage in net cage structures [3,4,5].

Sensors commonly used for fish detection typically include acoustic sensors and visual sensors. Acoustic sensors offer a wide detection range but are significantly influenced by environmental noise, and their relatively low resolution makes it difficult to acquire fine-grained fish features [6]. In comparison, visual sensors can capture high-resolution images, which are useful in inferring valuable information, such as size, semantic landmarks, and behaviors of fish.

**Figure 1:**
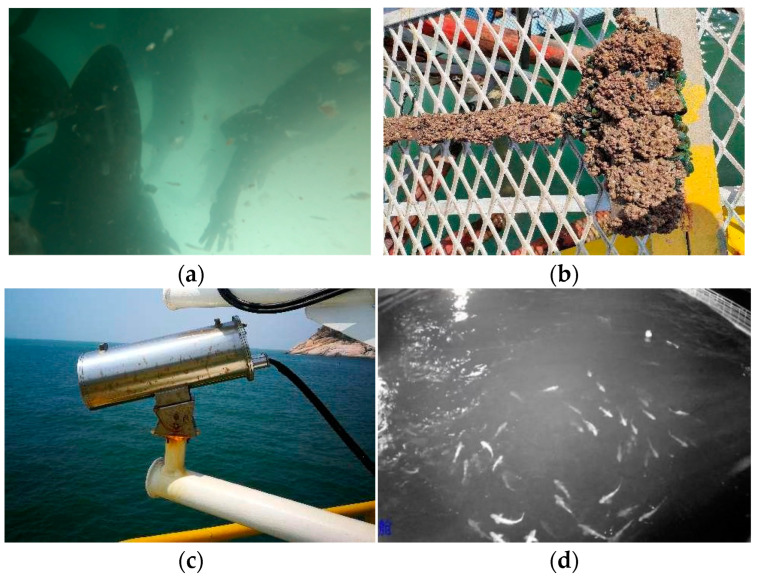
Comparison of different camera deployment methods: (**a**) image captured by underwater camera; (**b**) biofouled underwater camera; (**c**) above-water infrared camera; (**d**) image captured by above-water infrared camera.

Visual sensors can be deployed above water or underwater. In practice, underwater sensors struggle to capture clear images with a large field of view (Figure 1a) because of rapid light attenuation underwater and accumulated biofouling in sensors [7,8] (Figure 1b). On the contrary, cameras deployed above water do not encounter the aforementioned challenges and thus can offer a broad field of view for long-term, maintenance-free fish monitoring, which is considerably more suitable for monitoring fish species that prefer to swim at the water surface. Additionally, the total equipment and maintenance cost, which is within USD 2000 per year, is small compared to the annual profits of several hundred thousand dollars generated by a typical deep-sea net cage. Therefore, we adopt an above-water monitoring strategy using an above-water infrared camera to capture fish activity information for deep-sea aquaculture.

After obtaining images through visual sensors, the use of object detection algorithms enables the automatic detection of individual fish. Object detection is one of the fundamental tasks in computer vision. Due to the variations in target appearances, light conditions, and camera perspectives, robust and accurate object detection remains a challenging problem. Early object detection algorithms based on handcrafted features (such as the Viola–Jones detector [9], Histogram of Oriented Gradient (HOG) [10], and Deformable Part Model (DPM) [11]) have achieved good results on specific datasets but have struggled to handle more complex scenarios.

Since 2012, thanks to the development of deep convolutional neural networks (CNNs) [12,13,14] and the establishment of large image datasets [15,16,17], deep learning-based object detection algorithms have begun to dominate this field. These detection algorithms can be categorized into one-stage detectors (including Yolo [18], SSD [19], RetinaFace [20]) and two-stage detectors (including Fast R-CNN [21], Faster R-CNN [22], Cascade R-CNN [23]). They both utilize a CNN for robust and high-level image feature learning and extraction. The difference is that one-stage detectors directly use the feature vectors obtained by the feature extraction network for classification and regression, and two-stage detectors first generate a series of region proposals and then extract features from the proposed regions before performing classification and bounding box regression. Broadly speaking, one-stage models have better real-time performance and are easier to deploy on consumer electronics, while two-stage detectors achieve higher accuracy, especially for dense and small objects.

To improve the performance of object detection networks, researchers have proposed a variety of strategies. The Feature Pyramid Network (FPN) [24] is designed to improve the accuracy of detecting objects of varying sizes by effectively merging features from different scales, utilizing a top-down framework with lateral connections. By strategically combining 3 × 3 convolution kernels to simulate a larger kernel, a context module (CM) [25] has been devised, which achieves a large receptive field while maintaining a relatively small parameter size. Wang et al. [26] adopt 1D convolution to design a lightweight channel attention mechanism known as ECA-Net, which improves accuracy with only a slight increase in model complexity. Focal Loss [27] is introduced to address the negative impact of foreground–background class imbalance on the one-stage detector, which allows the accuracy of the one-stage detector to surpass that of the two-stage detector.

At the same time, numerous efforts have been made for the scenario of fish detection. Rosales et al. [28] and Muksit et al. [29] have respectively verified the effectiveness of Faster R-CNN and YOLO for fish detection. A two-stream Faster R-CNN architecture [30] by incorporating RGB and optical flow images as inputs is designed to detect fish, which uses both appearance and motion information to improve accuracy. Zeng et al. [31] propose an improved Faster R-CNN by adding an adversarial occlusion network that generates partially occluded feature maps as adversarial examples after the ROI pooling step, which helps improve the robustness of underwater fish detection. A novel two-stage method [32] for fish detection and classification is introduced. In this method, the object detection task is handled by a YOLO model, while the classification of fish is carried out by a CNN with squeeze-and-excitation (SE) architecture. Li et al. [33] refine the YOLOv5 model by integrating a Coordinate Attention (CA) mechanism alongside cross-stage partial networks, thereby improving the accuracy of fish object detection. Salman et al. [34] adopt background subtraction and optical flow methods to obtain fish regions as region proposals for R-CNN to address the issue of fish object detection. The comparison of our work with other fish detection research is illustrated in Table 1, where ‘AP50′ means the average precision at an intersection of union (IoU) threshold of 0.5.

In summary, researchers have proposed a large number of methods to solve the problem of general object detection and fish target detection, and have made great progress. However, how to select a suitable deep learning model for a new dataset is still unknown and difficult. The primary challenges lie in the fact that deep learning models operate as opaque “black boxes” with an extensive array of parameters, making it arduous to ascertain their performance through theoretical means alone. Furthermore, the model performance can be significantly influenced by variations in the training protocols, data augmentation strategies, and parameter initialization methods. Therefore, based on our fish dataset, this paper selects various deep learning models for comparative experiments.

The contributions of this paper are outlined as follows:

1. Based on the above-water infrared camera, a dataset for deep-sea aquaculture fish detection was constructed, comprising 400 images and 2830 individual fish.
2. Using our fish dataset, we compared the performances of Faster R-CNN and YOLOv5 and explored the influence of five different backbone networks (including VGG16, ResNet34, ResNet50, MobileNetV2, and EfficientNetB0), as well as different learning rates, feature extraction layers, and data augmentation strategies, on fish detection precision.
3. Furthermore, we investigated the impact on fish detection performance by integrating individual modules into Faster R-CNN, including the FPN, CM, ECA-Net, and the effect of the combined networks.

## 2. Materials and Methods

### 2.1. Data Gathering

**Figure 2:**
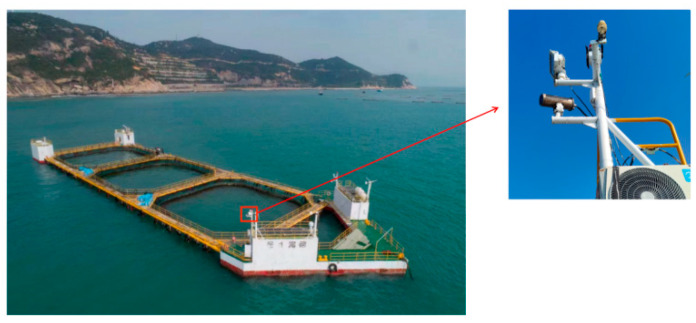
Camera mounted on a deep-sea truss-structure net cage.

Above-water infrared cameras have a wide field of view and are robust to changes in lighting and color, enabling effective fish monitoring even during nighttime or in low-light conditions. Therefore, our dataset was acquired from an infrared camera mounted on a truss-structure net cage, as shown in Figure 2. The camera used for data gathering was a Hikvision DS-2CD6626B-IZHRS produced by Hangzhou Hikvision Digital Technology Co., Ltd. in Hangzhou, China, and the captured images had a resolution of 1920 × 1080. In addition, images were gathered in July 2022 via continuous, around-the-clock recording in the maritime zone of Kuishan Island, Zhuhai, China. We then sampled nighttime images to build this dataset.

**Figure 3:**
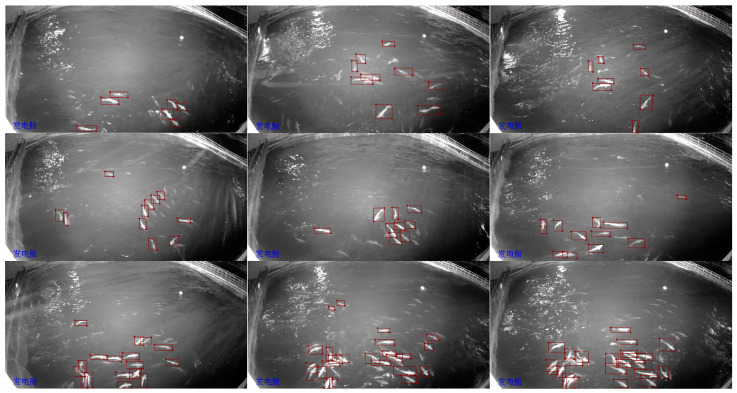
Labeled images. The blue Chinese characters in the lower left corner of the image are location markers, indicating that the camera used to capture this image is installed outside the power generation room.

A total of 400 images were sampled, and the fish targets within these images were annotated using Labelme v5.3.1, resulting in a total of 2830 fish being labeled. Some labeled images are displayed in Figure 3. It is important to note that, to detect clear and complete fish for subsequent length measurement and trajectory tracking, only fish that were visible and close to the water surface were labeled. Individuals with smaller exposed parts on the water surface were not annotated.

### 2.2. Network Modules for Fish Detection

This article adopted the Faster R-CNN as the base object detection framework and integrated additional modules such as the FPN, CM, and ECA-Net by leveraging various backbone networks including VGG16, ResNet34, ResNet50, MobileNetV2, and EfficientNetB0 to construct a range of detection networks. The details of each part are introduced separately below.

**Figure 4:**
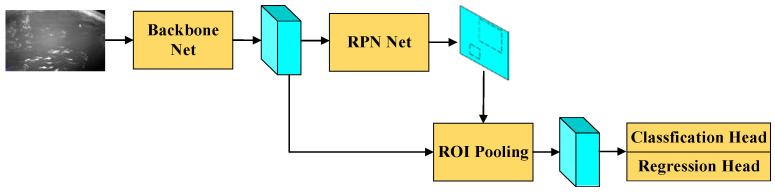
Faster R-CNN.

Faster R-CNN is a two-stage object detection network that has been applied across various fields. The processing steps for a given input image are as follows, and the network architecture is shown in Figure 4. First, the image is passed through a feature extraction network to obtain feature maps. Afterward, feature maps are processed by the region proposal network to obtain proposal regions, which include the shape parameters of candidate bounding boxes and the categories of objects within them. Then, with the region proposal as input, ROI pooling is applied to the feature maps to extract feature vectors of a uniform size. Finally, these feature vectors are fed into a fully connected network to perform classification and bounding box regression.

**Figure 5:**
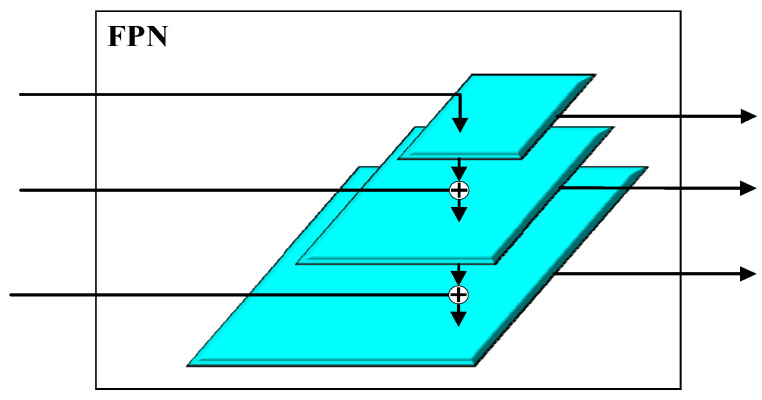
Feature Pyramid Network (FPN).

The FPN is used to fuse feature maps of different scales, maintaining both high-level semantic information and good resolution. The architecture of the FPN is shown in Figure 5. It is a multi-input, multi-output module. Its inputs come from the outputs of different layers of the backbone network, and its output consists of feature maps with multi-scale features aggregated. This module includes a top-down process for feature extraction, which is generally part of the backbone network, and a bottom-up process for multi-scale feature aggregation. The aggregation method involves upsampling the low-resolution feature maps and adding them to the high-resolution feature maps after a convolution operation.

**Figure 6:**
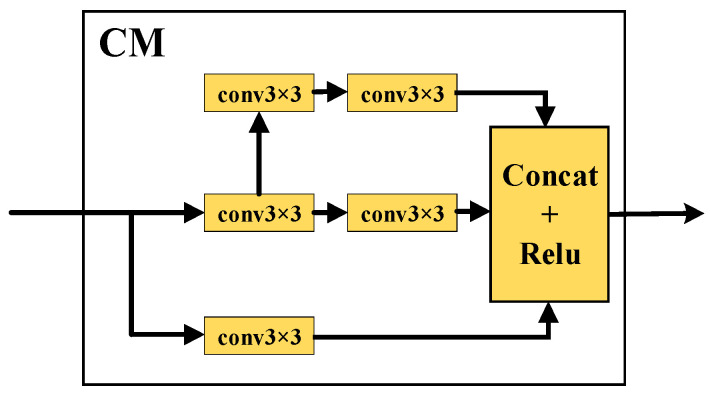
Context module (CM).

The context module is used to aggregate feature maps of different convolution kernel sizes, thereby increasing the receptive field of individual feature vectors. It is a single-input, single-output module. It takes feature maps as input and outputs aggregated feature maps, as shown in Figure 6. The key technique used in this module lies in replacing 5 × 5 or 7 × 7 convolution with a series of 3 × 3 convolution operations to improve the efficiency of weight usage.

**Figure 7:**
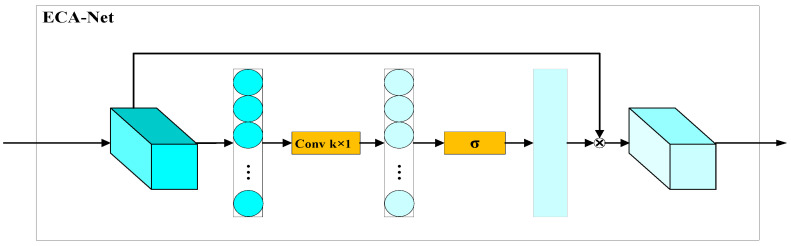
ECA-Net.

Channel attention is a method that re-weights feature maps with learned weights, which helps the network focus on more important features. The ECA-Net is a highly efficient and effective channel attention method that utilizes 1D convolution operations to compute the weight vector, thereby reducing the number of parameters and lessening the learning burden. The ECA-Net, displayed in Figure 7, is a single-input and -output module, which includes three steps. Firstly, global average pooling is applied to each channel of the input feature maps, transforming the H×W×C feature maps to 1×1×C vectors, where C represents the number of channels. Subsequently, the adaptive kernel size *k* of the one-dimensional convolution is calculated based on the input channel size. Convolution and activation are then performed using this kernel with a feature vector of 1×1×C, followed by normalization to obtain the channel weights. The feature maps are re-weighted using the channel weights and then passed out as the final output.

**Figure 8:**
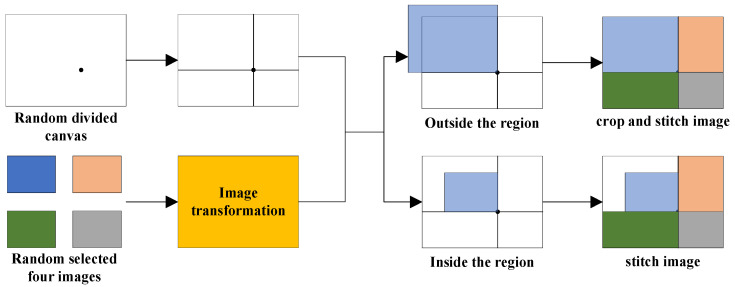
The steps of Mosaic data augmentation. The squares with the same color in different steps indicate the same image sample.

Data augmentation is an effective strategy for preventing overfitting in deep learning models by generating new training samples from existing datasets. We used the Mosaic technique for data augmentation, which creates new training samples by stitching together four transformed images. Figure 8 shows the steps of Mosaic. First, a random point is sampled on a canvas, which divides the canvas into four regions. Next, from the original dataset, four training images are randomly chosen and subjected to scaling and color space transformations. Finally, each transformed image is stitched onto a designated quadrant of the canvas, with any portions of the images that exceed the boundaries of their respective regions being cropped.

**Figure 9:**
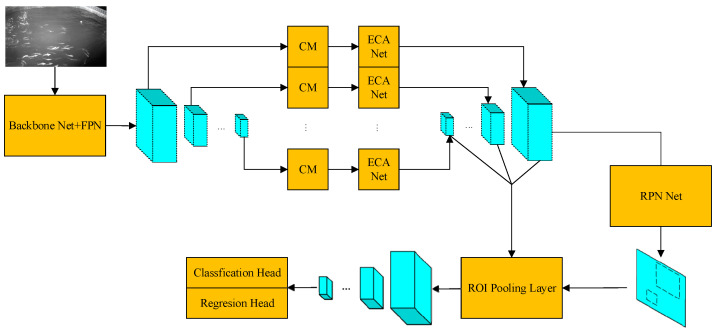
Faster R-CNN integrated with multiple modules.

Since the input and output of the FPN, CM, ECA-Net, and RPN Net modules are all multi-channel feature maps, and since the ROI pooling step of Faster R-CNN is consistent in processing both single and multiple channels, it is straightforward to form a combined network by connecting the input and output of these modules. We created the combined network as illustrated in Figure 9. The notation and combination order for the input and output feature maps of different modules are detailed in Table 2. More specifically, this integrated detection network processes the input image with the following steps. An input image first passes through the backbone and FPN for multi-scale feature extraction, yielding feature maps at different scales. Subsequently, each feature map undergoes further refinement and restructuring through a context module and an attention module to produce the final feature maps. The subsequent processing steps are consistent with those of Faster R-CNN, including proposal generation, ROI pooling, classification, and bounding box regression.

## 3. Experimental Setup and Results

In our experimental setup, we divide the dataset mentioned in Section 2.1 that comprises 400 images into a training set consisting of 225 images, a validation set of 75 images, and a testing set of 100 images. We use AP50 (average precision at IoU = 0.5), AP75 (average precision at IoU = 0.75), and AP (average precision at IoU = 0.50, 0.75, 0.95) to evaluate the precision of models, DT (detection time per image) to evaluate the efficiency of models, and PS (parameter size) to evaluate the size of models. The anchor box sizes of 32, 64, 128, 256, and 512 and aspect ratios of 0.5, 1.0, and 2.0 are selected as the anchor parameters for all Faster R-CNN-based networks. For YOLOv5, anchor parameters are obtained by its built-in clustering algorithm. The training and testing of all deep learning models are conducted on the same computer using the same software libraries.

### 3.1. Comparison Experiments between Faster RCNN and YOLOv5

This experiment compares the performance of the popular one-stage detection network YOLOv5 and the two-stage detection network Faster RCNN on our fish dataset. The backbone networks of YOLOV5 and Faster RCNN are CSPDarknet and VGG16, respectively. As shown in Table 3, the AP metric of Faster R-CNN is significantly higher than that of YOLOv5. However, YOLOv5 surpasses Faster R-CNN in terms of AR, and its average detection time per image (AT/s) is notably faster than Faster R-CNN.

### 3.2. Comparison Experiments under Different Learning Rates

In this experiment, we adopt Faster R-CNN with VGG16 as the backbone network for fish detection. We compare its performance under different learning rates and adaptive learning rates. Table 4 shows that the three constant learning rates and the adaptive learning rate have a minor impact on accuracy. Optimal results are achieved with a learning rate of 0.025. Higher learning rates can potentially lead to exploding gradients and NaN errors, which are not shown in this table.

### 3.3. Comparison Experiments under Different Backbone Networks

**Figure 10:**
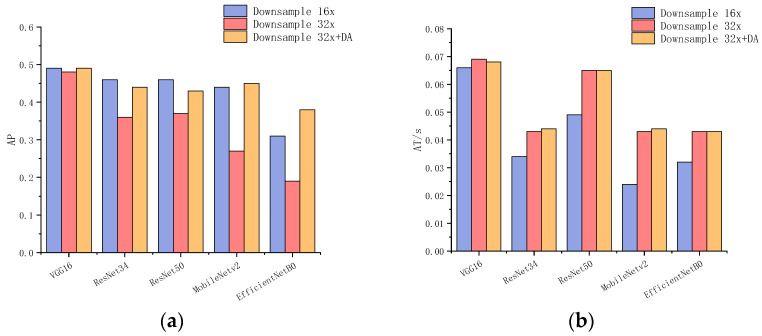
Bar chart for selected results from Table 3. The horizontal axis represents different network backbones, while the vertical axis represents the corresponding network’s AP metric or AT metric: (**a**) Comparison of AP metric. (**b**) Comparison of AT metric.

This study examines the influence of various backbone networks, feature maps derived from different layers of the same backbone network, and data augmentation techniques on the performance of Faster R-CNN. The backbone networks evaluated include VGG16, ResNet34, ResNet50, MobileNetv2, and EfficientNetB0. The results are presented in Table 5 and Figure 10, where “Lx” indicates the use of feature maps from the x-th layer as input for subsequent steps, “DR” refers to the accumulated downsampling rate, and “DA” implies the adoption of the Mosaic data augmentation method. Note that a layer with a downsampling rate of 2 produces output feature maps whose length and width are half of the input dimensions, and the accumulated downsampling rate of a specific layer is calculated by multiplying the downsampling rates of all previous layers. As a result, the output feature maps extracted from layers with the accumulated downsampling rates of 16 and 32 are different for different backbone networks, as shown in Table 5. Instead of “Lx”, we represent different layers using accumulated downsampling rates in the bar chart for simplicity, where the shallower layers have an accumulated downsampling rate of 16, and the deeper layers have an accumulated downsampling rate of 32.

The outcomes reveal that the choice of the backbone network and the layer of feature maps can impact detection accuracy. Notably, VGG16 with the L10 feature map achieves the best AP value while also maintaining a relatively small model size. Employing deeper feature maps fails to enhance detection accuracy with our dataset. Instead, it leads to a significant decline, particularly in ResNet34, ResNet50, MobileNetv2, and EfficientNetB0. Additionally, aside from VGG16, deeper feature layers show a substantial improvement in accuracy when data augmentation is applied. The primary reason for this phenomenon is that deeper neurons have larger receptive fields. However, given that a fish object has a smaller size, such large receptive fields may capture multiple fish or more of the varied background, which can lead to overfitting when the data are limited. The use of data augmentation helps to reduce this effect.

### 3.4. Comparison Experiments by Integrating Different Modules

**Figure 11:**
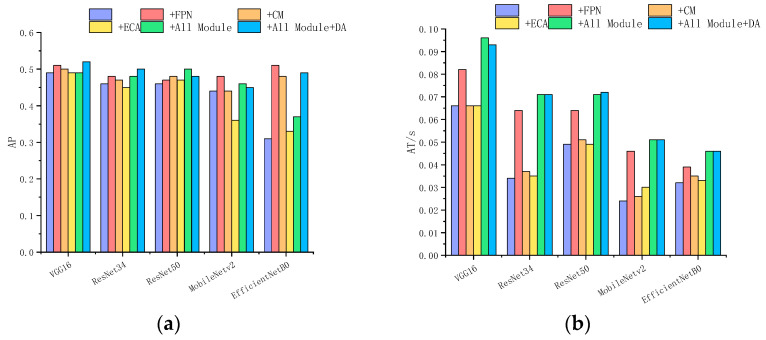
Bar chart for selected results from Table 4. The horizontal axis represents different network architectures and whether to use the data augmentation strategy, while the vertical axis represents the corresponding network’s AP metric or AT metric: (**a**) Comparison of AP metric. (**b**) Comparison of AT metric.

This experiment compares the detection accuracy of the Faster R-CNN network when integrating different modules. That is, within the Faster R-CNN framework shown in Figure 5, we integrate the FPN, CM, and ECA-Net. Furthermore, we validate the detection accuracy of the combined network shown in Figure 9. In this experiment, all FPN modules adopt a three-layer architecture, which means that the inputs consist of feature maps from three distinct layers of the backbone network. Specifically, for VGG16, the output of layers 4, 7, and 10 are selected as F_00, F_01, and F_02; for ResNet34, layers 7, 15, and 27 are selected; for ResNet50, layers 10, 22, and 40 are selected; for MobileNetV2, layers 4, 7, and 14 are incorporated; and for EfficientNetB0, layers 6, 9, and 16 are selected. The number of output channels for each scale of the FPN is 256, which means that the channel numbers for F10, F11, and F12 are 256. Similarly, the channel numbers for inputs and outputs of all CM and ECA-Net modules are 256, making the channel numbers for F20, F21, F22, F30, F31, and F32 the same. The final results are presented in Table 6 and Figure 11.

As can be seen from Table 4, after integrating the FPN module into different detection networks, all metrics improve, with a particularly significant increase in AP75. Among them, EfficientNetB0 achieves comprehensive improvements, with AP increasing from 0.31 to 0.51 and AP75 from 0.19 to 0.57. However, due to the integration of the FPN, which introduces a substantial number of parameters, there is a noticeable decrease in detection speed. The parameter size of Faster R-CNN with VGG16 and ResNet50 decreases after integrating the FPN module. This is because the FPN has 256 output channels. In contrast, before integrating the FPN, VGG16’s layer 10 and ResNet50’s layer 40 have 512 and 1024 output channels, respectively. Although the integration of the FPN module inherently increases the number of parameters, the significant reduction in channel count leads to a substantial decrease in the parameters for subsequent steps in Faster R-CNN, resulting in an overall reduction in the total parameter size.

After integrating CM, most accuracy metrics experience a slight improvement, with the accuracy of EfficientNetB0 showing a more noticeable improvement. At the same time, the weight of each detection network grows, which results in a decrease in the average detection speed.

After integrating ECA, the parameter sizes remain almost unchanged, with no significant impact on detection speed. Meanwhile, the accuracy improvements are limited, and some networks, including MobileNetv2 and ResNet34, even experience a decline.

The network that combined all modules shows an improvement in all accuracy metrics compared to the detection network without any integrated modules. However, compared to the network with only the FPN module, the combined network shows a decline in certain accuracy metrics, which does not meet the expected performance improvement. For instance, in the network with EfficientNetB0 and FPN, the AP value increases to 0.85, whereas after integrating all modules, the AP value only improves to 0.79. The reason is that combining all modules leads to a larger number of parameters and a deeper structure, which makes the model more complicated and easier to fit unsuitable features and noise, especially when the dataset has limited sample numbers. However, by augmenting the dataset with image transformations and cropping, the number of training samples increases, which allows the model to learn features that are invariant after such transformations. As these features exhibit enhanced generalization capabilities, they help improve the model performance when the model is too complicated for a limited dataset. We also evaluate the performance of the combined network after data augmentation. The results show an improvement in accuracy metrics. Notably, the AP75 scores of all combined networks, except EfficientNetB0, exceed the improvements achieved by integrating any single module.

## 4. Conclusions

To solve the problem of the long-term online automatic monitoring of fish for deep-sea aquaculture, we constructed a fish dataset based on an above-water infrared camera. At the same time, we carried out detailed comparative experiments on fish detection performance using the Faster R-CNN model and its variants. Our experimental results show that in our self-built dataset, the one-stage detection network Faster R-CNN outperforms the two-stage detection network YOLOv5 in terms of accuracy metrics. The features extracted by different backbone networks and the use of different layers as inputs for ROI pooling and RPN in Faster R-CNN affect both the accuracy and efficiency metrics. Additionally, a deeper network is not always superior, and an appropriate depth can achieve higher accuracy while maintaining a smaller parameter size. In most cases, integrating extra network modules will enhance the accuracy performance of the original network, with the FPN making a notable improvement. Moreover, data augmentation is an effective method to enhance model performance; it improves the detection accuracy of both the combined and original networks. Comparing all experimental results, Faster R-CNN with EfficientNetB0 and the FPN achieves excellent results with an AP value of 0.51 and an AP50 value of 0.85, which are competitive with the VGG16+All Module network trained with data augmentation. At the same time, it takes a significantly shorter detection time while maintaining the high AP values, making it a good choice for our application.

## Figures and Tables

**Table 1:** Comparison with other fish detection research.

Name	Method	Dataset	AP50
Faster R-CNN [28]	Faster R-CNN-based	underwater, fishpond	0.78
YOLO-Fish [29]	YOLO-based	underwater, wild environment	0.77
Multi-stream Faster R-CNN [30]	Faster R-CNN-based	underwater, wild environment	0.74
CME-YOLOv5 [33]	YOLO-based	underwater, hydropower stations, and fish breeding station	0.949
deep neural network-based hybrid motion learning [34]	R-CNN-based	underwater, wild environment	lack of data
Our work	Faster R-CNN-based	above-water, deep sea truss-structure net cage	0.85

**Table 2:** Notation and combination order for the input and output feature maps of different modules.

FPN		CM		ECA-Net	
Input	Output	Input	Output	Input	Output
F_00	F_10	F_10	F_20	F_20	F_30
F_01	F_11	F_11	F_21	F_21	F_31
F_02	F_12	F_12	F_22	F_22	F_32

**Table 3:** Results of comparison experiments between Faster R-CNN and YOLOv5.

Model	AP	AP50	AT(s)
YOLOv5	0.33	0.68	0.014
Faster RCNN	0.48	0.85	0.069

**Table 4:** Results of comparison experiments with different learning rates.

Learning Rate	AP	AP50	AP75
LR-0.005	0.48	0.85	0.48
LR-0.01	0.49	0.85	0.52
LR-0.025	0.50	0.86	0.52
LR-adaptive	0.48	0.85	0.49

**Table 5:** Results of the comparison experiments with varying backbone networks, feature layers, and data augmentation strategy.

Backbone	DR	AP	AP50	AP75	AT(s)	PS(M)
VGG16+L10	16	0.49	0.85	0.50	0.066	36.78
VGG16+L13	32	0.48	0.85	0.48	0.069	43.86
VGG16+L13+DA	32	0.49	0.84	0.54	0.068	43.86
ResNet34+L27	16	0.46	0.83	0.48	0.034	22.68
ResNet34+L33	32	0.36	0.74	0.28	0.043	50.43
ResNet34+L33+DA	32	0.44	0.79	0.42	0.044	50.43
ResNet50+L40	16	0.46	0.83	0.48	0.049	70.49
ResNet50+L49	32	0.37	0.76	0.30	0.065	165.23
ResNet50+L49+DA	32	0.43	0.78	0.42	0.065	165.23
MobileNetv2+L14	16	0.44	0.83	0.39	0.024	6.51
MobileNetv2+L19	32	0.27	0.70	0.14	0.043	82.35
MobileNetv2+L19+DA	32	0.45	0.82	0.41	0.044	82.35
EfficientNetB0+L16	16	0.31	0.73	0.19	0.032	13.91
EfficientNetB0+L18	32	0.19	0.54	0.05	0.043	84.14
EfficientNetB0+L18+DA	32	0.38	0.79	0.29	0.043	84.14

**Table 6:** Results of the comparative experiment by integrating different modules.

Feature Extraction Nodules	AP	AP50	AP75	AT(s)	PS(M)
VGG16	0.49	0.85	0.50	0.066	36.78
VGG16+FPN	0.51	0.85	0.57	0.082	24.13
VGG16+CM	0.50	0.85	0.54	0.066	38.99
VGG16+ECA	0.49	0.86	0.51	0.066	36.78
VGG16+All Module	0.49	0.85	0.52	0.096	25.79
VGG16+All Module+DA	0.52	0.86	0.60	0.63	0.093
ResNet34	0.46	0.83	0.48	0.034	22.68
ResNet34+FPN	0.48	0.85	0.53	0.064	24.55
ResNet34+CM	0.47	0.84	0.48	0.037	23.23
ResNet34+ECA	0.45	0.83	0.44	0.035	22.68
ResNet34+All Module	0.48	0.84	0.55	0.071	26.21
ResNet34+All Module+DA	0.50	0.82	0.57	0.61	0.071
ResNet50	0.46	0.83	0.48	0.049	538
ResNet50+FPN	0.47	0.83	0.48	0.064	193
ResNet50+CM	0.48	0.84	0.49	0.051	605
ResNet50+ECA	0.47	0.84	0.47	0.049	538
ResNet50+All Module	0.50	0.84	0.51	0.071	205
ResNet50+All Module+DA	0.48	0.82	0.55	0.59	0.072
MobileNetv2	0.44	0.83	0.39	0.024	6.51
MobileNetv2+FPN	0.48	0.84	0.50	0.046	16.85
MobileNetv2+CM	0.44	0.83	0.40	0.026	6.58
MobileNetv2+ECA	0.36	0.79	0.27	0.030	6.51
MobileNetv2+All Module	0.46	0.83	0.47	0.051	18.51
MobileNetv2+All Module+DA	0.45	0.85	0.54	0.63	0.051
EfficientNetB0	0.31	0.73	0.19	0.032	13.91
EfficientNetB0+FPN	0.51	0.85	0.57	0.039	19.22
EfficientNetB0+CM	0.48	0.85	0.51	0.035	14.23
EfficientNetB0+ECA	0.33	0.74	0.22	0.033	13.91
EfficientNetB0+All Module	0.37	0.79	0.25	0.046	20.88
EfficientNetB0+All Module+DA	0.49	0.84	0.53	0.63	0.046

## Data Availability

The raw data supporting the conclusions of this article will be made available by the authors on request.
